# Acute High-Dose and Chronic Lifetime Exposure to Alcohol Consumption and Differentiated Thyroid Cancer: T-CALOS Korea

**DOI:** 10.1371/journal.pone.0151562

**Published:** 2016-03-17

**Authors:** Yunji Hwang, Kyu Eun Lee, Elisabete Weiderpass, Young Joo Park, Young Jun Chai, Hyungju Kwon, Do Joon Park, BeLong Cho, Ho-Chun Choi, Daehee Kang, Sue K. Park

**Affiliations:** 1 Department of Preventive Medicine, Seoul National University College of Medicine, Seoul, Korea; 2 Department of Biomedical Science, Seoul National University Graduate School, Seoul, Korea; 3 Cancer Research Institute, Seoul National University, Seoul, Korea; 4 Department of Surgery, Seoul National University Hospital & College of Medicine, Seoul, Korea; 5 Division of Surgery, Thyroid Center, Seoul National University Cancer Hospital, Seoul, Korea; 6 Department of Community Medicine, Faculty of Health Sciences, University of Tromsø, The Arctic University of Norway, Tromsø, Norway; 7 Department of Research, Cancer Registry of Norway, Oslo, Norway; 8 Department of Medical Epidemiology and Biostatistics, Karolinska Institutet, Stockholm, Sweden; 9 Department of Genetic Epidemiology, Folkhälsan Research Center, Helsinki, Finland; 10 Department of Internal Medicine, Seoul National University College of Medicine, Seoul, Korea; 11 Department of Surgery, Seoul National University Boramae Medical Center, Seoul, Korea; 12 Breast and Thyroid Cancer Center, Ewha Womans University College of Medicine, Seoul, Korea; 13 Department of Family Medicine, Health Promotion Center for Cancer survivor, Seoul National University Hospital, Seoul, Korea; 14 Advanced Institutes of Convergence Technology, Seoul National University, 145 Gwanggyo-ro, Yeongtong-gu, Suwon–si, Gyeonggi-do, Korea; 15 Department of Family Medicine, Healthcare System Gangnam Center, Seoul National University Hospital, Seoul, Korea; Peking Union Medical College, CHINA

## Abstract

**Background:**

This study evaluated the effects of acute high-dose and chronic lifetime exposure to alcohol and exposure patterns on the development of differentiated thyroid cancer (DTC).

**Methods:**

The Thyroid Cancer Longitudinal Study (T-CALOS) included 2,258 DTC patients (449 men and 1,809 women) and 22,580 healthy participants (4,490 men and 18,090 women) who were individually matched by age, gender, and enrollment year. In-person interviews were conducted with a structured questionnaire to obtain epidemiologic data. Clinicopathologic features of the patients were obtained by chart reviews. Odds ratios (ORs) and 95% confidence intervals (95%CI) were estimated using conditional regression models.

**Results:**

While light or moderate drinking behavior was related to a reduced risk of DTC, acute heavy alcohol consumption (151 g or more per event or on a single occasion) was associated with increased risks in men (OR = 2.22, 95%CI = 1.27–3.87) and women (OR = 3.61, 95%CI = 1.52–8.58) compared with never-drinkers. The consumption of alcohol for 31 or more years was a significant risk factor for DTC for both men (31–40 years: OR = 1.58, 95%CI = 1.10–2.28; 41+ years: OR = 3.46, 95%CI = 2.06–5.80) and women (31–40 years: OR = 2.18, 95%CI = 1.62–2.92; 41+ years: OR = 2.71, 95%CI = 1.36–5.05) compared with never-drinkers. The consumption of a large amount of alcohol on a single occasion was also a significant risk factor, even after restricting DTC outcomes to tumor size, lymph node metastasis, extrathyroidal extension and TNM stage.

**Conclusion:**

The findings of this study suggest that the threshold effects of acute high-dose alcohol consumption and long-term alcohol consumption are linked to an increased risk of DTC.

## Introduction

There has been a significant increase in thyroid cancer worldwide over the last three decades [1]. Enhanced detection of small tumors, using advanced diagnostic imaging technologies and new screening practices, has contributed to this reported increase [2, 3]. However, the incidence of large tumors has also increased, suggesting the contribution of other environmental or lifestyle factors [1]. In Korea, thyroid cancer was the most common cancer in 2012, with an age-standardized prevalence rate of 358.7 per 100,000 and an age-standardized incidence rate of 62.5 per 100,000 [4]. An increase in the alcohol drinking rate from 1998 to 2005 was observed for both men (57.0% to 66.1%) and women (23.4 to 34.5%), based on the Korean National Health and Nutrition Examination Survey data [5]. Considering these continuous increases, alcohol consumption and thyroid cancer are serious public health concerns in Korea [5, 6].

While an association between drinking alcohol and the risk of developing cancer has been proposed, inconsistent results have been reported for thyroid cancer [7]. Previous studies, including case-control studies [8], cohort studies [9] and pooled analysis [10], have reported that alcohol consumption is inversely associated with thyroid cancer risk. However, other studies have reported an absence of a significant association [7, 11–14]. These conflicting results have been based on data reported by studies of less than 500 thyroid cancer patients [7–9, 11], a study restricted only to postmenopausal females [12], and studies that have failed to evaluate the effect modifiers [10, 13]. Abnormal functioning of the hypothalamic-pituitary-thyroid (HPT) axis has been observed in chronic alcoholics, indicating the potential involvement of chronic ethanol exposure in thyroid hormone metabolism [15]. Binge alcohol exposure rather than the total volume of alcohol consumption, increased the risk of metabolic syndrome [16], obesity [17], and mortality caused by oropharyngeal and esophageal cancers [18] in Koreans. However, few epidemiologic studies have focused on the harmful effects of long-term alcohol consumption or heavy alcohol consumption per event on the development of thyroid cancer or the risk of thyroid cancer with unfavorable clinicopathologic features.

The aim of this study was to confirm the possible relationship between alcohol consumption and differentiated thyroid cancer (DTC) risk, accounting for drinking status and duration and amount of alcohol consumption. Data from the Thyroid Cancer Longitudinal Study (T-CALOS), which was performed in Korea, were analyzed. Associations were further examined in patient subgroups divided according to clinicohistologic characteristics, including histologic type, tumor size, lymph node metastasis, extrathyroidal extension, BRAF (V600E) mutation and TNM staging.

## Materials and Methods

### Study subjects: selection of cases and controls

T-CALOS is an on-going, multidisciplinary study involving the collection of data from thyroid cancer patients and healthy controls [19]. Between April 2010 and April 2014, 2,529 newly diagnosed and pathologically confirmed thyroid cancer patients were recruited after undergoing thyroidectomy at the Department of Surgery, Seoul National University Hospital, Korea. After excluding those without information on drinking status, we included a total of 2,257 DTC patients (448 men and 1,809 women). We selected controls from the large, healthy examinee cohort of the Korean Genome Cohort Study (KoGeS). Out of a pool of controls examined from 2004 to 2012 (n = 170,082), we excluded those subjects with a history of any type of cancer at enrollment (n = 795) or missing information for drinking status (n = 1,552). We matched individuals at a 1:10 (cases: controls) ratio by age (no more than a 5-year difference), sex and enrollment year (no more than a 5-year difference), and selected 22,570 controls for this analysis (4,490 men and 18,090 women). This matching was performed at random using statistical software (SAS, Version 9.3, SAS Institute, Cary, NC), and %GMATCH macro was used to perform greedy matching based on the given matching variables and the ratio of the cases to the controls, without any discrimination or interference by the authors [20]. Finally, we included 2,257 cases and 22,570 controls in these analyses.

### Data collection

The subjects completed an in-person interview, during which their demographic information, lifestyle factors and medical history were obtained using a structured questionnaire. All of the interviewers and interviewees were blinded to the purpose of this study. Drinking status was classified into two groups of “never-drinkers” and “ever-drinkers”. The questionnaire included questions about the consumption of various types of alcoholic beverages, including beer, wine, gin, and four types of Korean traditional beverages (soju, rice wine, refined rice wine and fruit wine). The subjects selected one of 8 options for drinking frequency (almost never, once per month, 2–3 times per month, once per week, 2–3 times per week, 4–6 times per week, once per day and more than twice per day) for each type of beverage based on the alcohol consumption patterns of the previous year. The volume was defined for each type of drink as follows: beer (200 ml), wine (90 ml), hard liquor (30 ml), soju (50 ml), rice wine (250 ml), refined rice wine (50 ml) and fruit wine (50 ml). The amount (g) of alcohol consumption was calculated using an ethanol intensity of 0.79 and beverage-specific alcohol content (5% for beer, 12% for wine, 40% for hard liquor, 20% for soju, 6% for rice wine, 15% for refined rice wine and 15% for fruit wine). Alcohol consumption per event for the ever-drinkers was estimated as the total amount consumed at a single occasion and was categorized into 5 groups (0–25 g, 26–50 g, 51–100 g, 101–150 g, and 151 g or more) or 3 groups (0–50 g, 51–150 g and 151 g or more). We further classified ever-drinkers by the drinking duration (0–10 years, 11–20 years, 21–30 years, 31–40 years, and 41+ years) and using generous cutoff values (0–20 years, 21–30 years, and 31+ years) because of the small number of subjects evaluated in subgroup analyses. After quantifying the reported alcohol consumption of the individuals, we divided the total alcohol consumption by the frequency of alcohol consumption to determine alcohol consumption per day (g/day). Binge drinking was defined as excessive alcohol consumption on a single occasion (5 drinks for men and 4 drinks for women), with a standard drink equal to 14 g of alcohol.

For all cases, comprehensive reviews of the electronic medical records were conducted to determine clinicopathologic features. We used the AJCC/UICC TNM staging system (7th edition), which is based on age at diagnosis, tumor size, presence of an extrathyroidal extension, lymph node metastasis and distant metastasis [21]. Genetic testing for the BRAF (V600E) mutation was performed on papillary thyroid carcinoma (PTC) patients. The deoxyribonucleic acid (DNA) isolation and BRAF (V600E) mutation analysis methods and the details of all of the protocols followed for the T-CALOS are described elsewhere [19, 22].

### Statistical analysis

Descriptive statistical analyses were performed to compare the DTC cases with their matched healthy controls. Differences in numerical and categorical variables were evaluated with the t-test and chi-square test, respectively. Conditional logistic regression models were used to compute odds ratios (ORs) and 95% confidence intervals (95%CIs) for both the univariate and multivariate models. To control for potential confounders, ORs and 95%CIs were adjusted in the model for education level (high school graduation: yes, no, or unknown), marital status (single, married, or unknown), smoking (never, past, or current), regular exercise (yes, no, or unknown), and history of chronic diseases, including hypertension and dyslipidemia (diagnosed by a medical doctor: never, ever, or unknown). No evidence of multicollinearity was found based on diagnostic analysis. The dependent variables included tumor size, lymph node metastasis, extrathyroidal extension, TNM stage, age at diagnosis and V600E BRAF mutation status in the three nominal categories (the controls, the cases with low aggressive tumor features and the cases with high aggressive tumor features). The associations between drinking-related predictors and DTC risk were presented as ORs and 95%CIs based on polychotomous logistic regression models adjusted for matching variables (age, sex and enrollment year) and education level, marital status, smoking, regular exercise, and history of chronic diseases, including hypertension and dyslipidemia. We included only the PTC patients and their matched controls in BRAF (V600E) mutation analyses.

To identify interactions, a multiplicative interaction model was used based on the likelihood ratio test, for which the main factor of alcohol exposure and the variables of education level, marital status, smoking and chronic diseases were included. Sensitivity analyses were conducted by restricting the age at diagnosis of the cases (40, 45 and 50 years old) to confirm the robustness of the results. We calculated p-trends for the dose-response associations by assigning increasing scores for the levels of the categorical variables, and these scores were used in the fully adjusted regression models. The p-heterogeneity for the comparisons of the associations in each group was calculated using Cochran’s Q statistics. A p value of less than 0.05 was considered significant based on the 2-sided test. Statistical analyses were conducted with SAS software program (Version 9.4, SAS Institute, Cary, NC).

### Ethical approval

All subjects in this study voluntarily participated and provided their written informed consent upon enrollment. The entire study protocol was approved by the Institutional Review Board of Seoul National University Hospital (IRB No. C-1001-067-307).

## Results

### General characteristics

The general characteristics of the 24,827 total subjects, including the 2,257 DTC patients (cases) and 22,570 healthy participants (controls), are summarized in S1 Table. Eighty percent of the subjects were women, and the average ages (in years) of the cases (49.9 for the men and 50.4 for the women) and controls (50.5 for the men and 50.8 for the women) did not significantly differ, indicating that the individual matching was appropriate. The cases were more likely to be educated and have chronic diseases (hypertension and dyslipidemia) compared with the healthy controls. The patient group was less likely to be married, smoke, and exercise regularly (*p* <0.05) compared with the control group (S1 Table). The number of postmenopausal women did not significantly differ between the cases (877 women, 50.2%) and controls (9087 women, 50.3%) (p-value = 0.93). The variables that were determined to be statistically significant were considered to be confounding factors and were adjusted for in multivariate regression analyses.

### Alcohol intake and DTC

As observed in Table 1, no evidence of an association between drinking itself and DTC risk was observed (men: OR = 1.12; 95%CI = 0.85–1.47; women: OR = 1.10, 95%CI = 0.99–1.22). A trend in the J-shaped curves was detected for an association between alcohol intake on a single occasion and increased DTC risk, indicating a decreased DTC risk for light to moderate alcohol consumption and an elevated risk for heavy alcohol consumption. Heavy alcohol consumption per event (151+ g) was a significant risk factor for DTC in men (OR = 2.21, 95%CI = 1.27–3.85) and women (OR = 3.61, 95%CI = 1.52–8.58) compared with the never-drinkers (Table 1). Sensitivity analysis conducted to confirm the associations revealed that the results were robust after we restricted the subjects by the cutoff ages of 40, 45 and 50 years old (S2 Table). DTC risk was found to be altered by excessive alcohol intake (21 g/day: OR = 1.49, 95%CI = 1.08–2.06) and binge drinking (4 or more drinks per event: OR = 1.98, 95%CI = 1.48–2.64) in women compared with never-drinkers (data not shown). Stratification by histologic type revealed that the association between PTC risk and alcohol consumption on one occasion (151+g, men: OR = 2.17, 95%CI = 1.23–3.83; women: OR = 3.56, 95%CI = 1.50–8.46) was consistent with the results for total DTC, as shown in Table 2. A trend of increasing follicular thyroid cancer (FTC) risk in association with alcohol intake on one occasion was observed, but the results were not statistically significant (Table 2).

**Table 1:** Alcohol consumptions and differentiated thyroid cancer in men and women; T-CALOS April 2010–April 2014.

	Men			Women			
	Case (N = 448)	Control (N = 4,480)	OR (95%CI)^1^	Case (N = 1,809)	Control (N = 18,090)	OR (95%CI)^1^	*p*-heterogeneity^2^
**Drinking status**							
Never	78	828	1.00 (Reference)	1,111	11,322	1.00 (Reference)	
Ever	370	3,652	1.12 (0.85–1.47)	698	6,768	1.10 (0.99–1.22)	0.884
**Alcohol intake (g) per event**							
Never	78	828	1.00 (Reference)	1,111	11,322	1.00 (Reference)	
0–25	46	636	0.74 (0.50–1.11)	310	3,832	0.86 (0.75–0.99)	0.502
26–50	44	619	0.77 (0.52–1.15)	119	1,389	0.90 (0.74–1.10)	0.503
51–100	170	1,599	1.25 (0.92–1.69)	109	937	1.29 (1.03–1.60)	0.911
101–150	35	338	1.18 (0.76–1.84)	13	70	2.27 (1.23–4.21)	0.091
151+	20	108	2.21 (1.27–3.85)	7	24	3.61 (1.52–8.58)	0.353
*p*-trend^2^			*0*.*005*			*0*.*044*	
**Duration (years)**							
Never	78	828	1.00 (Reference)	1,111	11,322	1.00 (Reference)	
0–10	10	172	0.46 (0.22–0.98)	121	1,853	0.71 (0.58–0.86)	0.282
11–20	107	1174	0.84 (0.59–1.19)	245	3,020	0.81 (0.69–0.94)	0.853
21–30	106	1295	0.81 (0.58–1.13)	195	1,436	1.43 (1.21–1.69)	0.003
31–40	95	767	1.57 (1.09–2.26)	61	308	2.18 (1.62–2.92)	0.183
41+	43	224	3.44 (2.05–5.78)	13	45	2.71 (1.40–5.24)	0.571
*p*-trend^2^			*<0*.*001*			*<0*.*001*	

Abbreviations: OR = odds ratio; 95% CI = 95% confidence interval.

^1^. Conditional logistic regression models adjusted for education level, marital status, smoking, regular exercise, and history of hypertension and dyslipidemia.

^2^. *p-*trend was calculated for dose-response associations, and *p*-heterogeneity was calculated to compare of the ORs and their 95% CI for the men and women.

**Table 2:** Alcohol consumptions and thyroid cancer stratified by histologic types (papillary and follicular thyroid cancer); T-CALOS April 2010–April 2014.

	Men				Women			
	PTC^1^		FTC^1^		PTC^1^		FTC^1^	
	Case	OR (95% CI)^2^,^3^	Case	OR (95% CI) ^2^,^3^	Case	OR (95% CI) ^2^,^3^	Case	OR (95% CI) ^2^,^3^
**Alcohol intake (g) per event**								
Never	76	1.00 (Reference)	2	1.00 (Reference)	1,079	1.00 (Reference)	32	1.00 (Reference)
0–50	83	0.72 (0.52–1.02)	7	1.54 (0.28–8.50)	413	0.86 (0.76–0.98)	16	1.16 (0.60–2.27)
51–150	197	1.23 (0.91–1.67)	8	1.83 (0.32–10.28)	118	1.34 (1.09–1.66)	4	*1*.*44 (0*.*44–4*.*74)*^6^
151+^4^	19	2.17 (1.23–3.83)	1	5.36 (0.34–85.68)	7	3.56 (1.50–8.46)	0	
*p*-trend^5^		*0*.*004*		*0*.*578*		*0*.*232*		
**Duration (years)**								
Never	76	1.00 (Reference)	2	1.00 (Reference)	1,079	1.00 (Reference)	32	1.00 (Reference)
0–20	115	0.82 (0.58–1.16)	2	0.26 (0.03–2.43)	351	0.76 (0.66–0.87)	15	1.07 (0.52–2.20)
21–30	100	0.81 (0.58–1.15)	6	1.70 (0.24–11.92)	190	1.42 (1.20–1.69)	5	*1*.*50 (0*.*60–3*.*78)*^6^
31+^4^	125	1.75 (1.23–2.48)	13	13.27 (1.11–158.28)	72	2.38 (1.81–3.12)	2	
*p*-trend^5^		*0*.*006*		*0*.*018*		*<0*.*001*		*0*.*458*

Abbreviations: PTC = papillary thyroid cancer; FTC = follicular thyroid cancer; OR = odds ratio; 95% CI = 95% confidence interval.

^1^. Subjects with missing information for alcohol intake per event with FTC (5 men and 4 women) and PTC (50 men and 136 women) were identified.

^2^. Conditional logistic regression models adjusted for education level, marital status, smoking, regular exercise, and history of hypertension and dyslipidemia.

^3^. No significant *p*-heterogeneity detected in the comparison of the ORs for PTC and FTC.

^4^. Due to insufficient power in analyses of the FTC patients and their matched controls, the highest cutoff values were changed to a drinking duration of 21+ years and 51 g of alcohol intake per event.

^5^. A *p-*trend was calculated for dose response associations.

^6^. ORs were calculated after merging the two cells of (51+ for alcohol intake amounts (g) per event and 21+ for the duration (years) of drinking) due to the limited number of cases.

### Drinking duration and DTC

Threshold effects of drinking duration were also observed (Table 1). The DTC risk was generally lower for those subjects reporting 10 years or less of alcohol consumption (men: OR = 0.46, 95%CI = 0.22–0.98; women: OR = 0.71, 95% CI = 0.58–0.86). In contrast, those reporting 31–40 years of alcohol consumption showed a 2-fold increased risk of DTC (men: OR = 1.57, 95%CI = 1.09–2.26; women: OR = 2.18, 95%CI = 1.62–2.92) compared with the never-drinkers. A 3-fold increased risk of DTC was observed in subjects reporting 41 years or more of alcohol consumption (men: OR = 3.44, 95%CI = 2.05–5.78; women: OR = 2.71, 95%CI = 1.40–5.24) (*p*-trend<0.05) compared to the never-drinkers (Table 1). Our results showed that women were more susceptible to drinking duration compared to men (*p*-heterogeneity for 21–30 years of drinking = 0.003), whereas both men and women showed similar patterns for the association of alcohol consumption per event with drinking duration (Table 1). The general trend of these results was consistent even after restricting the subjects to those who were older than 40, 45 and 50 years (S2 Table). In addition, the number of years of alcohol consumption was associated with PTC risk (31+ years, men: OR = 1.75, 95%CI = 1.23–2.48; women: OR = 2.38, 95%CI = 1.81–3.12) as well as male FTC risk (OR = 13.27, 95%CI = 1.11–158.28), as shown in Table 2. There were no significant differences in the PTC or FTC risk according to the *p*-heterogeneity values.

### Alcohol consumption and DTC by clinicopathologic features

Associations of alcohol consumption and DTC risk were classified according to patient age at diagnosis and clinicopathologic features, such as tumor size, lymph node metastasis, extrathyroidal extension, BRAF mutation and TNM staging. Alcohol consumption per event (≥151 g) was associated with a 2.2-fold increased risk of DTC with a tumor size of 1 cm or greater (OR = 2.23, 95%CI = 1.09–4.53) and a 1.1-fold increased risk of DTC with a tumor size of smaller than 1 cm (OR = 1.11, 95%CI = 0.29–2.11) compared to the never-drinkers, as shown in Table 3. There was also a positive association between long-term drinking (31 years or longer) and DTC with a tumor size of 1 cm or greater (OR = 2.16, 95%CI = 1.57–2.96) and DTC with a tumor size of 1 cm or smaller (OR = 1.79, 95%CI = 1.41–2.28) compared to the never-drinkers (data not shown). The effects of alcohol intake per event (≥151 g) were consistent after the cases were stratified according to the presence of lymph node metastasis, extrathyroidal extension, and advanced TNM stage (Table 3). The BRAF mutation statuses of the PTC patients (BRAF^V600E^: OR = 2.89, 95%CI = 1.72–4.86; BRAF^wt^: OR = 2.59, 95%CI = 1.03–6.54) and the ages at diagnosis of the DTC patients (<45 years old: OR = 1.79, 95%CI = 0.72–4.48; ≥45 years old: OR = 1.88, 95%CI = 1.02–3.47) were also estimated in comparison with the never-drinkers (Table 3). The differences in the clinicopathologic features between the subgroups according to drinking duration were not statistically significant.

**Table 3:** Alcohol consumptions and differentiated thyroid cancer by clinicopathologic features, T-CALOS April 2010–April 2014.

	Case^1^	OR (95% CI)^2^	Case^1^	OR (95% CI) ^2^	Case^1^	OR (95% CI) ^2^	Case^1^	OR (95% CI) ^2^
		Tumor size≤1cm		Tumor size>1cm		LN metastasis (-)		LN metastasis (+)
**Alcohol intake (g) per event**								
Never	833	1.00 (Reference)	334	1.00 (Reference)	716	1.00 (Reference)	385	1.00 (Reference)
0–50	369	0.80 (0.69–0.92)	140	0.81 (0.65–1.00)	313	0.85 (0.73–0.98)	156	0.68 (0.56–0.83)
51–150	222	1.09 (0.89–1.32)	97	1.18 (0.89–1.57)	155	1.08 (0.87–1.35)	142	1.14 (0.89–1.46)
151+	14	1.11 (0.59–2.11)	11	2.23 (1.09–4.53)	11	1.22 (0.60–2.47)	15	1.87 (0.99–3.50)
*p*-trend^4^		*0*.*469*		*0*.*570*		*0*.*822*		*0*.*746*
**Duration (years)**								
Never	833	1.00 (Reference)	334	1.00 (Reference)	716	1.00 (Reference)	385	1.00 (Reference)
0–20	337	0.76 (0.65–0.88)	135	0.84 (0.67–1.05)	270	0.79 (0.67–0.93)	172	0.72 (0.58–0.88)
21–30	210	0.93 (0.78–1.12)	81	0.90 (0.68–1.18)	170	1.00 (0.82–1.22)	107	0.83 (0.65–1.06)
31+	132	1.80 (1.41–2.29)	76	2.14 (1.56–2.95)	107	1.95 (1.50–2.54)	79	1.89 (1.39–2.57)
*p*-trend^4^		*0*.*077*		*0*.*012*		*0*.*011*		*0*.*258*
		ETE (-)		ETE (+)		TNM stage I		TNM stage II-IV
**Alcohol intake (g) per event**								
Never	468	1.00 (Reference)	682	1.00 (Reference)	817	1.00 (Reference)	300	1.00 (Reference)
0–50	254	0.98 (0.83–1.16)	249	0.69 (0.59–0.81)	393	0.80 (0.70–0.92)	99	0.75 (0.58–0.95)
51–150	148	1.28 (1.01–1.63)	164	0.97 (0.78–1.21)	236	1.08 (0.89–1.32)	72	1.17 (0.84–1.62)
151+	11	1.51 (0.75–3.08)	14	1.39 (0.74–2.62)	19	1.39 (0.77–2.51)	7	1.87 (0.81–4.33)
*p*-trend^4^		*0*.*119*		*0*.*051*		*0*.*538*		*0*.*767*
**Duration (years)**								
Never	468	1.00 (Reference)	682	1.00 (Reference)	817	1.00 (Reference)	300	1.00 (Reference)
0–20	232	0.92 (0.77–1.10)	227	0.67 (0.56–0.79)	415	0.81 (0.70–0.94)	54	0.53 (0.39–0.73)
21–30	145	1.15 (0.92–1.43)	145	0.79 (0.64–0.97)	217	0.93 (0.78–1.12)	72	1.08 (0.80–1.44)
31+	86	2.15 (1.61–2.87)	117	1.73 (1.34–2.23)	103	1.81 (1.40–2.36)	83	1.90 (1.38–2.62)
*p*-trend^4^		*<0*.*001*		*0*.*564*		*0*.*138*		*0*.*013*
		Age at diagnosis <45		Age at diagnosis ≥45		BRAF^wt^ in PTC^3^		BRAF^V600E^ in PTC^3^
**Alcohol intake (g) per event**								
Never	293	1.00 (Reference)	896	1.00 (Reference)	349	1.00 (Reference)	763	1.00 (Reference)
0–50	213	0.79 (0.62–0.99)	306	0.69 (0.60–0.79)	156	0.91 (0.74–1.12)	326	0.83 (0.72–0.96)
51–150	151	1.23 (0.91–1.67)	176	1.21 (0.97–1.50)	73	1.12 (0.82–1.54)	229	1.49 (1.23–1.81)
151+	10	1.79 (0.72–4.48)	17	1.88 (1.02–3.47)	6	2.59 (1.03–6.54)	20	2.89 (1.72–4.86)
*p*-trend^4^		*0*.*735*		*0*.*389*		*0*.*280*		*0*.*017*
**Duration (years)**								
Never	293	1.00 (Reference)	896	1.00 (Reference)	349	1.00 (Reference)	763	1.00 (Reference)
0–20	319	0.73 (0.58–0.91)	164	0.58 (0.48–0.70)	123	0.69 (0.54–0.87)	324	0.82 (0.70–0.95)
21–30	101	2.83 (2.05–3.90)	200	1.23 (1.02–1.48)	89	1.41 (1.07–1.85)	192	1.18 (0.98–1.43)
31+	1	0.19 (0.01–61.69)	211	2.08 (1.68–2.59)	51	2.87 (1.91–4.34)	136	2.03 (1.58–2.61)
*p*-trend^4^		*<0*.*001*		*<0*.*001*		*<0*.*001*		*<0*.*001*

Abbreviations: OR = odds ratio; 95% CI = 95% confidence interval; LN metastasis = lymph node metastasis; BRAF^wt^ in PTC = negative for BRAF (V600E) by mutation testing; BRAF^V600E^ in PTC = positive for BRAF (V600E) by mutation testing; PTC = papillary thyroid cancer; DTC = differentiated thyroid cancer; ETE (-) = absence of extrathyroidal extension; ETE (+) = presence of extrathyroidal extension.

^1^. Subjects with missing information for alcohol intake per event with FTC (5 men and 4 women) and PTC (50 men and 136 women) were identified. DTC patients with information on tumor size (<1 cm = 1,567 cases; ≥1 cm = 644 cases), LN metastasis (No = 1,311 cases; Yes = 764 cases) and TNM staging (stage I = 1,606 cases; stage II = 41 cases; stage III = 406 cases; and stage IV = 79 cases), and ETE (No = 977 cases; Yes = 1,198 cases) and the controls were included.

^2^. Polychotomous logistic regression models (controls vs. less advanced cases; and controls vs. more advanced cases) were adjusted for matching variables (age, sex and enrollment year), education level, marital status, smoking, regular exercise, and history of chronic diseases, including hypertension and dyslipidemia.

^3^. ORs and 95%CIs were calculated for the PTC patients with information on BRAF mutation status (2,092 cases) and their matched controls using conditional logistic regression models adjusted for education level, marital status, smoking, regular exercise, and history of chronic diseases, including hypertension and dyslipidemia (diagnosed by a medical doctor: never, ever and unknown).

^4^. A *p-*trend was calculated for dose response associations.

## Discussion

The results of this large-scale case-control study that included 2,258 DTC patients and 22,580 healthy controls suggest that light to moderate drinking is associated with a reduced risk of DTC in men and women. However, there were noticeable threshold effects of acute high-dose and chronic lifetime exposure to alcohol on increasing the DTC risk. Our results were consistent, even after the grouping of cases according to clinicopathologic features. These associations support the importance of the consideration of alcohol consumption and drinking duration when defining a group at high risk of thyroid cancer in Korea.

The consumption of large amounts of alcohol may lead to increased risks of various cancers. The International Agency for the Research of Cancer (IARC) has classified alcohol as a group 1 carcinogen, with the highest level of carcinogenic effects [23]. A recent review paper has reported that the amount of alcohol consumption is linked to oral, pharyngeal, laryngeal and esophageal cancers (cancers of the head and neck) as well as liver and breast cancers [24]. However, whether these effects influence the thyroid gland in addition to their carcinogenic potentials are unknown. Additionally, plausible mechanisms have been suggested in which alcohol accelerates oxidative stress, DNA damage, and hormonal changes [24].

We found that women were more vulnerable than men to the duration of drinking. Although the underlying mechanism of the differences in susceptibility between men and women has not been elucidated, the effects of sex hormones on ethanol metabolism can be the explanation [25]. First, the androgens in men possibly increased alcohol dehydrogenase activity and enzymes responsible for the related pathways [26]. Second, women had a higher blood level of alcohol with a same amount of alcohol drinking [27], which may be the reason for their increased development of alcohol-related organ damage [25]. In addition, a low gastric alcohol dehydrogenase (ADH) activities and a slow alcohol elimination rate (AER) in the women have been observed in women, and the studies suggested a smaller organ size in women compared to that in men might related to differences in ethanol pharmacokinetics [25, 28]. These factors might result in a longer exposure time of thyroid tissue to alcohol per year of drinking duration and promote alcohol-induced adverse effects in women.

Few studies have assessed the associations of drinking and DTC, considering clinicopathologic features. We found consistent effects of drinking on DTC with a large tumor size or advanced TNM stage, suggesting the presence of a potential link between drinking and DTC with a poor prognosis. One recent study proposed that alcohol consumption may increase thyroid cancer risk by increasing thyroid-stimulating hormone (TSH) levels, thereby stimulating related hormones and mitotic activity and altering tumor susceptibility [29]; however, the aim of this study was not to determine the association between drinking and thyroid cancer risk. The BRAF (V600E) mutation has been associated with unfavorable clinicopathologic characteristics in patients with PTC in previous studies [22, 30]. The possible effects of drinking and BRAF mutations on colon cancer risk have been examined [31, 32], although, no related studies of drinking and thyroid cancer risk have been performed. Therefore, further evidence is needed to elucidate an associated biological mechanism that includes the following four components: alcohol consumption, thyroid cancer, clinicopathologic features and DNA mutations.

Previous studies have presented an inverse relationship between drinking and thyroid cancer. Based on the NIH-AARP Diet and Health Study, Meinhold et al. observed that consuming 2 or more drinks per day reduced thyroid cancer risk in a combined sample of men and women (RR = 0.57, 95%CI = 0.36–0.89) compared with never-drinkers [9]. However, these results were not statistically significant in subgroups classified by gender or histologic type, reflecting the insufficient power of the study due to the small range of alcohol consumption, particularly in women and FTC cases [9]. In a recent pooled analysis that included 1,003 cases, consuming alcohol daily or more often was found to be associated with a decreased thyroid cancer risk (≥7 drinks per week: hazard ratio (HR) = 0.72, 95%CI = 0.58–0.90) compared with never-drinkers [10]. Guignard et al. performed a case-control study (332 cases and 412 controls) and reported that there was no significant alteration in thyroid cancer risk due to alcohol consumption in men (>10 drinks per week: OR = 0.92, 95%CI = 0.24–3.45) or women (>10 drinks per week: OR = 0.32, 95%CI = 0.05–1.95) [11]. Furthermore, no significant results related to alcohol consumption and thyroid cancer were reported for the Women’s Health Initiative, which included 331 thyroid cancer patients (≥7 drinks per week: HR = 0.66, 95%CI = 0.44–1.01; ≥4 g/day: HR = 0.79, 95%CI = 0.60–1.05) compared to non-drinkers [12]. In comparison to these studies, opposite or insignificant associations were consistently observed in our results. Given these previous findings, we acknowledge that a small amount of alcohol consumption up to a certain threshold level for both acute and chronic lifetime exposure may have preventive effects on thyroid cancer. However, this study provides new evidence on the threshold effects of the number of years of alcohol consumption (40 years) and the amount of alcohol consumed per event (150 g per instance), using extended cutoff values that have not been assessed in previous studies. The conflicting results of previous studies may also be explained by geographic differences among these studies, including 5 prospective studies of individuals from the United States [10] and New Caledonia [11] and of postmenopausal women [12], all of whom have different patterns of alcohol consumption and, ethnicity and cultural factors compared to individuals from Korea.

The strengths of the present study are as follows. First, T-CALOS is one of the few studies that includes over 2,000 thyroid cancer cases, including both men and women. Therefore, our sample size of male thyroid cancer and FTC cases was large. In addition, our study was well-designed and conducted using a standardized research protocol, a comprehensive epidemiologic questionnaire and clinicopathologic information. Therefore, we included standard units or quantifiable amounts of alcohol and accounted for various confounding factors in statistical analyses. The limitations of our study include the fact that we used a case-control study design, which may have introduced recall bias. Although we collected self-reported information for the exposure variables, we attempted to maximize the accuracy and compliance of our interviews by using a structured questionnaire and providing continuous training for our interviewers. Due to the small number of female FTC patients who were drinkers, it was not possible to examine and confirm the associations of the threshold effects in this subgroup. Moreover, biomarkers related to thyroid function and BRAF mutation testing were only obtained for the thyroid cancer patients.

In conclusion, we observed a reduction in thyroid cancer risk in association with decreased alcohol consumption (25 g or less) per event and a drinking duration of less than 10 years compared with never-drinkers. However, the risk was increased above the threshold level in association with both the number of years of alcohol consumption (30 years old more) and alcohol consumption per event (151 g or more) for both men and women. The reason for the rapid increase in thyroid cancer in Korea in recent years is unknown. Because alcohol consumption is an important modifiable dietary risk factor for cancer, high-risk groups of individuals, such as long-term drinkers or those who consume excessive amounts of alcohol, should be carefully monitored and provided cancer prevention guidelines. Considering the increasing trends in adolescent drinking and binge drinking among young people, our findings highlight the need for further studies on alcohol consumption patterns and chronic lifetime exposure to alcohol in the development of thyroid cancer and the increased risk of thyroid cancer with unfavorable clinicopathologic features. Our results may contribute to the current understanding of the etiology of thyroid cancer, and thereby facilitate the development of preventative strategies.

## Supporting Information

S1 Table Comparison of the general characteristics of cases and controls, T-CALOS April 2010–April 2014. (DOCX)

S2 Table Alcohol consumptions and differentiated thyroid cancer in men and women restricted by the age group, T-CALOS April 2010–April 2014. (DOCX)

## Abbreviations

T-CALOS: Thyroid Cancer Longitudinal Study
DTC: differentiated thyroid cancer
OR: odds ratio
HR: hazard ratio
95%CI: 95% confidence interval
HPT: hypothalamic-pituitary-thyroid
KoGeS: Korean Genome Cohort Study
PTC: papillary thyroid carcinoma
FTC: follicular thyroid cancer
IARC: The International Agency for the Research of Cancer
TSH: thyroid-stimulating hormone
