# Supplementary material for: Acute High-Dose and Chronic Lifetime Exposure to Alcohol Consumption and Differentiated Thyroid Cancer: T-CALOS Korea
Source: PLoS One. 2016 Mar 17;11(3):e0151562. doi: 10.1371/journal.pone.0151562 (PMC4795733; doi:10.1371/journal.pone.0151562)
Supplement: S1 Table — (DOCX) [file pone.0151562.s001.docx]

**S1 Table. Comparison of the general characteristics of cases and controls, T-CALOS April 2010–April 2014**

|  | Cases (N=2,257) | Controls (N=22,570) | *p*-value^1^ |
| --- | --- | --- | --- |
| Age (*Mean (SE))* | 50.29 (0.21) | 50.71 (0.06) | 0.055 |
| BMI (*Mean (SE))* | 23.45 (0.07) | 23.59 (0.02) | 0.049 |
|  |  |  |  |
|  | *n (%)* | *n (%)* |  |
| Gender |  |  |  |
| Men | 448 (19.85) | 4,480 (19.85) | 1.000 |
| Women | 1,809 (80.15) | 18,090 (80.15) |  |
|  |  |  |  |
| Education level |  |  |  |
| Less than high school | 361 (15.99) | 5,636 (24.97) | <0.001 |
| High school or more | 1,889 (83.70) | 16,794 (74.41) |  |
| Unknown | 7 (0.31) | 140 (0.62) |  |
|  |  |  |  |
| Marital status |  |  |  |
| Not married | 137 (6.07) | 902 (4.00) | <0.001 |
| Married | 2,115 (93.71) | 21,644 (95.90) |  |
| Unknown | 5 (0.22) | 24 (0.11) |  |
|  |  |  |  |
| Smoking |  |  |  |
| Never | 1,877 (83.16) | 18,008 (79.79) | <0.001 |
| Past | 256 (11.70) | 1,932 (8.56) |  |
| Current | 124 (5.49) | 1,953 (8.65) |  |
| Unknown | 0 (0.00) | 677 (3.00) |  |
|  |  |  |  |
| Regular exercise |  |  |  |
| No | 1,279 (56.67) | 11,258 (49.88) | <0.001 |
| Yes | 974 (43.15) | 11,299 (50.06) |  |
| Unknown | 4 (0.18) | 13 (0.06) |  |
|  |  |  |  |
| History of hypertension |  |  |  |
| No | 1,739 (77.05) | 18,799 (83.29) | <0.001 |
| Yes | 516 (22.86) | 3,770 (16.70) |  |
| Unknown | 2 (0.09) | 1 (0.00) |  |
|  |  |  |  |
| History of dyslipidemia |  |  |  |
| No | 1,871 (82.90) | 20,255 (89.74) | <0.001 |
| Yes | 375 (16.61) | 2,311 (10.24) |  |
| Unknown | 11 (0.49) | 4 (0.02) |  |
|  |  |  |  |
| History of diabetes mellitus |  |  |  |
| No | 2,115 (93.71) | 21,342 (94.56) | 0.092 |
| Yes | 142 (6.29) | 1,228 (5.44) |  |

Abbreviation: SE=Standard Error; BMI=Body Mass Index.

1. Comparison of healthy controls and thyroid cancer patients, and the *p*-values were based on *t*-test for continuous variables and Pearson’s Chi-square test for categorical variables.
