# Supplementary material for: Acute High-Dose and Chronic Lifetime Exposure to Alcohol Consumption and Differentiated Thyroid Cancer: T-CALOS Korea
Source: PLoS One. 2016 Mar 17;11(3):e0151562. doi: 10.1371/journal.pone.0151562 (PMC4795733; doi:10.1371/journal.pone.0151562)
Supplement: S2 Table — (DOCX) [file pone.0151562.s002.docx]

**S2 Table. Alcohol consumptions and differentiated thyroid cancer in men and women restricted by the age group, T-CALOS April 2010–April 2014**

|  | Men | | | Women | | |  |
| --- | --- | --- | --- | --- | --- | --- | --- |
|  | Case | Control | OR (95% CI)^1^ | Case | Control | OR (95% CI)^1^ | *p*-heterogeneity^2^ |
| *Age>40 years old* |  |  |  |  |  |  |  |
| **Alcohol intake (g) per event** |  |  |  |  |  |  |  |
| Never | 58 | 648 | 1.00 (Reference) | 964 | 9687 | 1.00 (Reference) |  |
| 0-50 | 70 | 969 | 0.80 (0.55-1.16) | 320 | 3818 | 0.87 (0.76-1.00) | 0.670 |
| 51-150 | 142 | 1398 | 1.23 (0.87-1.73) | 76 | 704 | 1.19 (0.92-1.54) | 0.871 |
| 151+^4^ | 15 | 84 | 2.12 (1.12-4.02) | 7 | 17 | 4.65 (1.88-11.54) | 0.165 |
| *p*-trend^5^ |  |  | *0.020* |  |  | *0.689* |  |
|  |  |  |  |  |  |  |  |
| **Duration (years)** |  |  |  |  |  |  |  |
| Never | 58 | 648 | 1.00 (Reference) | 964 | 9687 | 1.00 (Reference) |  |
| 0-20 | 36 | 539 | 0.59 (0.37-0.94) | 179 | 3158 | 0.58 (0.49-0.68) | 0.929 |
| 21-30 | 99 | 1202 | 0.81 (0.56-1.17) | 194 | 1366 | 1.43 (1.20-1.69) | 0.006 |
| 31+^4^ | 138 | 991 | 1.90 (1.33-2.71) | 73 | 348 | 2.20 (1.68-2.89) | 0.516 |
| *p*-trend^5^ |  |  | <0.001 |  |  | <0.001 |  |
|  |  |  |  |  |  |  |  |
| *Age>45 years old* |  |  |  |  |  |  |  |
| **Alcohol intake (g) per event** |  |  |  |  |  |  |  |
| Never | 52 | 574 | 1.00 (Reference) | 821 | 8274 | 1.00 (Reference) |  |
| 0-50 | 56 | 834 | 0.74 (0.49-1.11) | 230 | 2769 | 0.87 (0.74-1.01) | 0.461 |
| 51-150 | 114 | 1106 | 1.26 (0.87-1.83) | 54 | 494 | 1.23 (0.91-1.67) | 0.919 |
| 151+^4^ | 13 | 66 | 2.47 (1.23-4.94) | 3 | 12 | 3.43 (0.94-12.46) | 0.660 |
| *p*-trend^5^ |  |  | *0.013* |  |  | *0.897* |  |
|  |  |  |  |  |  |  |  |
| **Duration (years)** |  |  |  |  |  |  |  |
| Never | 52 | 574 | 1.00 (Reference) | 821 | 8274 | 1.00 (Reference) |  |
| 0-20 | 24 | 327 | 0.69 (0.41-1.17) | 129 | 2122 | 0.63 (0.52-0.77) | 0.772 |
| 21-30 | 64 | 952 | 0.63 (0.41-0.95) | 116 | 1076 | 1.09 (0.88-1.34) | 0.022 |
| 31+^4^ | 138 | 988 | 1.82 (1.26-2.62) | 73 | 341 | 2.23 (1.70-2.94) | 0.375 |
| *p*-trend^5^ |  |  | 0.001 |  |  | 0.004 |  |
|  |  |  |  |  |  |  |  |
| *Age>50 years old* |  |  |  |  |  |  |  |
| **Alcohol intake (g) per event** |  |  |  |  |  |  |  |
| Never | 45 | 471 | 1.00 (Reference) | 662 | 6609 | 1.00 (Reference) |  |
| 0-50 | 43 | 668 | 0.67 (0.43-1.06) | 156 | 1889 | 0.85 (0.71-1.02) | 0.348 |
| 51-150 | 82 | 782 | 1.27 (0.84-1.92) | 34 | 292 | 1.30 (0.89-1.91) | 0.922 |
| 151+^4^ | 6 | 49 | 1.57 (0.61-4.03) | 3 | 7 | 6.02 (1.50-24.06) | 0.117 |
| *p*-trend^5^ |  |  | *0.079* |  |  | *0.887* |  |
|  |  |  |  |  |  |  |  |
| **Duration (years)** |  |  |  |  |  |  |  |
| Never | 45 | 471 | 1.00 (Reference) | 662 | 6609 | 1.00 (Reference) |  |
| 0-20 | 12 | 214 | 0.57 (0.29-1.12) | 85 | 1397 | 0.63 (0.50-0.80) | 0.774 |
| 21-30 | 25 | 533 | 0.45 (0.26-0.78) | 48 | 666 | 0.70 (0.51-0.95) | 0.172 |
| 31+^4^ | 132 | 973 | 1.58 (1.07-2.32) | 71 | 330 | 2.18 (1.65-2.89) | 0.177 |
| *p*-trend^5^ |  |  | *0.002* |  |  | *0.101* |  |

Abbreviation: OR=Odds Ratio; 95% CI=95% Confidential Interval.

1. Conditional logistic regression models adjusted for education level, marital status, smoking, regular exercise, history of hypertension and dyslipidemia.

2. *p-*trend was calculated for dose-response associations, and *p*-heterogeneity was calculated to compare of ORs and their 95% CI in men and women.
